# Correlation study between osteoporosis and hematopoiesis in the context of adjuvant chemotherapy for breast cancer

**DOI:** 10.1007/s00277-017-3184-6

**Published:** 2017-11-23

**Authors:** Frédérica Schyrr, Anita Wolfer, Jérôme Pasquier, Anne-Laure Nicoulaz, Olivier Lamy, Olaia Naveiras

**Affiliations:** 1 Laboratory of Regenerative Hematopoiesis, Swiss Institute for Experimental Cancer Research (ISREC) & Institute of Bioengineering, École Polytechnique Fédérale de Lausanne (EPFL), Lausanne, Switzerland; 2 Department of Oncology, University Hospital Lausanne (CHUV), Lausanne, Switzerland; 3 Institute of Social and Preventive Medicine (IUMSP), University Hospital, Lausanne, Switzerland; 4 Base de données des Centres Interdisciplinaires en Oncologie – CINO, CHUV, Lausanne, Switzerland; 5 Service de médecine interne, département de médecine, CHUV, Lausanne, Switzerland; 6 Centre des Maladies Osseuses (CMO), Département de l’Appareil Locomoteur, CHUV, Lausanne, Switzerland; 7 Service d’Hématologie, Département d’Oncologie, CHUV, Lausanne, Switzerland

**Keywords:** Hematopoiesis, Osteoporosis, Osteoblast, T-score, TBS, Trabecular bone score, Chemotherapy, Breast cancer, Bone marrow microenvironment, Bone marrow adipocytes, Stress hematopoiesis, Neutrophil, Platelet, Anemia, Febrile neutropenia, Graded toxicity of chemotherapy

## Abstract

This retrospective study attempts to establish if a correlation exists between osteoporosis and hematopoiesis before and after adjuvant chemotherapy in the context of non-metastatic breast cancer. Osteoporosis is interpreted both as a direct marker of osteoblastic decline and as an indirect marker of increased bone marrow adiposity within the hematopoietic microenvironment. Patients from the “Centre du Sein” at CHUV (Centre Hospitalier Universitaire Vaudois) undergoing adjuvant chemotherapy were included in this study. Evolution of blood counts was studied in correlation with the osteoporosis status. Toxicity of chemotherapy was coded according to published probability of febrile neutropenia. One hundred forty-three women were included: mean age 52.1 ± 12.5 years, mean BMI (body mass index) 24.4 ± 4.1. BMD (bone mineral density) scored osteoporotic in 32% and osteopenic in 45%. Prior to chemotherapy, BMD was positively correlated with neutrophil (*p* < 0.001) and thrombocyte (*p* = 0.01) count; TBS (trabecular bone score) was not correlated with blood count. After the first cycle of chemotherapy, an increase of one point in TBS correlated with a decrease of 57% on the time to reach leucocyte nadir (*p* = 0.004). There was a positive correlation between BMD and risk of infection (*p* < 0.001). Our data demonstrates an association between osteoporosis and lower blood counts in a younger cohort than previously published, extending it for the first time to neutrophil counts in females. Our results suggest that the healthier the bone, the earlier the lowest leucocyte count value, prompting further research on this area.

## Introduction

### Hematopoietic stem cell niche

Hematopoiesis is the process by which precursor and mature blood cells are produced by hematopoietic stem cells (HSCs) within the adult bone marrow. In adults, the hematopoietic marrow resides within the axial skeleton and the proximal epiphysis of the femur and the humerus. In these areas, the marrow consists of 10–90% fat depending on age. In fact, it is estimated that the bone marrow adipose tissue represents around 1 to 1.5 kg in a healthy adult [1, 2].

The HSC niche, located within the bone marrow, is critical for the maintenance of the HSC. The niche is composed of different cells including osteoblasts, mesenchymal stromal cells, perivascular cells, and adipocytes [3, 4].

Osteoblasts have a regulatory role in the HSC niche and support maintenance of the most primitive HSCs. Osteoblasts are a key element for the myeloid lineage as murine osteoblasts have been shown to produce G-CSF (granulocyte colony-stimulating factor), M-CSF (macrophage colony-stimulating factor), GM-CSF (granulocyte-macrophage colony-stimulating factor), IL-1 (interleukin-1), IL-6 (interleukin-6) among other cytokines that support HSC proliferation [5]. At the same time, osteoblasts also produce inhibitory molecules, such as osteopontin, that limit hematopoietic replication and have an overall supporting role in HSC long-term maintenance [5–10].

Adipocytes are important components of the hematopoietic microenvironment. Although long considered passive space fillers within the bone marrow, they also secret cytokines with mixed hematopoetic activity, such as neuropilin-1, adiponectin, and leptin. In mice, fully differentiated adipocytes have been shown to have a negative regulatory effect on HSC proliferation in the context of stress hematopoiesis and post-transplantation aplasia, while both in mice and human they support the survival of the most primitive hematopoietic stem cells [11–15].

### Osteoporosis

Osteoporosis is a skeletal disorder characterized by compromised bone strength and microarchitectural deterioration. The bone mineral density (BMD) is evaluated by dual-energy X-ray absorptiometry (DXA). The result of the BMD is given as a T-score relative to normal values from a pool of a healthy 25-year-old population. Osteoporosis is defined by a T-score below − 2.5 standard deviations (SD) [16]. The trabecular bone score (TBS) is a gray-level textural index, derived from lumbar spine DXA images, that is correlated with parameters reflecting bone microarchitecture [17]. TBS provides skeletal information that is not captured with standard BMD measurements. A low TBS is consistently associated with an increased risk of prevalent and incident fractures. Therefore, bone composition can be partially deducted from T-score and TBS.

Osteoporosis, at the cellular level, can be explained by an imbalanced activity of osteoblasts and osteoclasts. This process results in an increase of the bone turnover and a trabecular bone loss which in turn results in increased adipocyte content [16, 18–23]. Thus, osteoporosis is interpreted here both as a direct marker of osteoblastic decline and as an indirect marker of increased bone marrow adiposity.

### Osteoporosis and breast cancer

Several studies have suggested an inverse relationship between BMD and breast cancer incidence [24]. Moreover, cancer treatment that reduces estrogens levels affects negatively BMD in premenopausal women [25]. Interestingly, BMD loss in premenopausal women during 6 months’ adjuvant systemic chemotherapy seems to be independent of changes in ovarian function [26]. Postmenopausal women with breast cancer who received adjuvant chemotherapy had lower BMD than those who did not [27]. It is estimated that postmenopausal women with breast cancer lose 2–3 fold more BMD, in comparison with healthy postmenopausal women [28].

We thus decided to test whether there is a correlation between osteoporosis and hematopoiesis upon stress hematopoiesis before and after adjuvant chemotherapy, in the context of a breast cancer cohort.

## Methods

### Cohort of breast cancer patients:

All women treated for localized breast cancer at the Centre Hospitalier Universitaire Vaudois (CHUV), Lausanne, Switzerland, between August 2008 and July 2015 were evaluated for this retrospective study. The women were included if they (1) had a non-metastatic breast cancer at the time of diagnosis, (2) belonged to the “Centre du Sein” database with no document attesting disagreement to share their data for research projects, and (3) received chemotherapy. BMD was evaluated by DXA before or at onset of adjuvant endocrine therapy.

The criteria for exclusion were absence of available BMD measurement 3 years prior or following the diagnosis, absence of available laboratory values following chemotherapy, any prior chemotherapy, BMI > 35, history of hematological disorders or of active hematological disease, active second malignancy concomitant to breast cancer, or moderate to severe renal impartment. Some patients received endocrine therapy after completion of the chemotherapy treatment. As blood counts are not influenced by endocrine therapy, this was not an exclusion criteria.

### Bone parameters

BMD was measured on the femoral neck, total femur, and lumbar spine and TBS was measured at lumbar spine L1 to L4. According to the BMD values, osteoporosis was defined if any of the three locations obtained a T-score < − 2.5 SD, osteopenia as a T-score between − 1.0 and − 2.5 SD, normal as a T-score > − 1.0 SD [16].

The following normal range for TBS values was used: TBS > 1.310 was considered to be normal, TBS between 1.230 and 1.310 was considered to be consistent with partially degraded microarchitecture, and TBS < 1.230 defined degraded microarchitecture.

### Collected data

We collected the identification code of the patients, date of birth, diagnosis, date of diagnosis, type of chemotherapy, starting date of chemotherapy, T-score, and TBS values.

To study hematological recovery, all available blood counts between 1 week prior to the first cycle of chemotherapy and the first day of the second cycle of chemotherapy were collected. The incidence of infection between the first day of chemotherapy and the first day of the second cycle of chemotherapy was also recorded.

Confounding factors taken into account for the multivariate analysis were the age at the time of treatment, the use of G-CSF, and the type of chemotherapy, which was graded according to hematological toxicity.

To code the toxicity of the chemotherapy, we took advantage of previous literature discussing the recommended use of G-CSF during chemotherapy for breast cancer [29]. Zielinski et al. created a table with the associated risk of febrile neutropenia for each standard adjuvant breast cancer chemotherapy regime (Table 1). The risk is given as the percentage of patients who will present febrile neutropenia without G-CSF. The clinical trials data of each chemotherapy regime were used to generate the table with the risk of febrile neutropenia.

**Table 1:** Frequency of adjuvant chemotherapy regimes in our cohort. The corresponding risk of febrile neutropenia according to Zielinski et al. was used to code the toxicity of the chemotherapy regime as a continuous variable

Chemotherapy	Risk of febrile neutropenia during cycle of chemotherapy, (%) (adapted from ref. [21])	Number of patients (*n* = 143), (%)	G-CSF treatment administered during the 1st cycle of chemotherapy (*n* = 143), (%)
Doc-Car	13	3 (2.1)	3 (100)
FEC-Doc	11	42 (29.4)	7 (17)
FEC	6	2 (1.4)	0 (0)
AC-Pac	5	1 (0.7)	1 (100)
Cap-Doc	5	1 (0.7)	0 (0)
EC	5	4 (2.8)	3 (75)
ECP	5	15 (10.5)	9 (60)
FEC-Pac	5	1 (0.7)	0 (0)
TC	5	66 (46.2)	24 (36)
AC	3	2 (1.4)	0 (0)
Paclitaxel	2	6 (4.2)	0 (0)

*Doc-Car* docetaxel–carboplatine–trastuzumab, *FEC-Doc* 5-fluorouracil–epirubicin–cyclophosphamide–docetaxel, *FEC* 5-fluorouracil–epirubicin–cyclophosphamide, *AC-Pac* doxorubicin–cyclophosphamide–paclitaxel, *Cap-Doc* capecitabine-docetaxel, *EC* epirubicin–cyclophosphamide, *ECP* epirubicin–cyclophosphamide–paclitaxel, *FEC-Pac* 5-fluorouracil–epirubicin–cyclophosphamide–paclitaxel, *TC* docetaxel–cyclophosphamide, *AC* doxorubicin–cyclophosphamide

We used this reference to code the hematological toxicity of the different chemotherapy using the published risk of febrile neutropenia for each chemotherapy regime as a continuous variable to adjust for intensity of hematological toxicity in our multivariate analysis.

### Response variables

To establish the response variables, we collected both the date and the absolute value for neutrophil, leucocyte, and thrombocyte nadir. We calculated the difference between the starting count and the neutrophil, leucocyte, and thrombocyte count at nadir, as well as the rate of infection.

### Statistical analysis

For the analysis of the blood count before chemotherapy, linear regressions were performed.

The univariate analysis was performed using GraphPad Prism version 7.0a for Mac OS X, GraphPad Software, San Diego California USA, www.graphpad.com. The multivariate analysis was performed using R (version 3.3.1).

For the three variables “date for neutrophil, leucocyte and thrombocyte nadir,” both a Poisson regression and a linear regression were performed and both gave similar results. For the six variables “value of neutrophil, leucocyte and thrombocyte nadir” and “difference between the starting count and the neutrophil, leucocyte and thrombocyte count at nadir,” a linear regression was performed. For the variable “infection,” a logistic regression was performed. Each regression was performed four times changing the explicative variable: TBS score continuous, TBS score categorical, T-score continuous, and T-score categorical. Each regression was adjusted for the variables age, G-CSF, and toxicity of chemotherapy. Some variables showed missing values. Complete case analyses were performed.

The quality control of the regressions was done using residual versus fitted predicted values.

### Ethical committee

All procedures were in accordance with the ethical standards of the responsible committee on human experimentation and in accordance with the 1975 Helsinki declaration as revised in 2008. The local ethical commission approved the study (CER-VD, Lausanne, Switzerland). For this type of study, specific consent was not required. Patients having provided a document attesting disagreement to share their medical data for research projects were excluded.

## Results

### Characteristics of the cohort

**Fig. 1:**
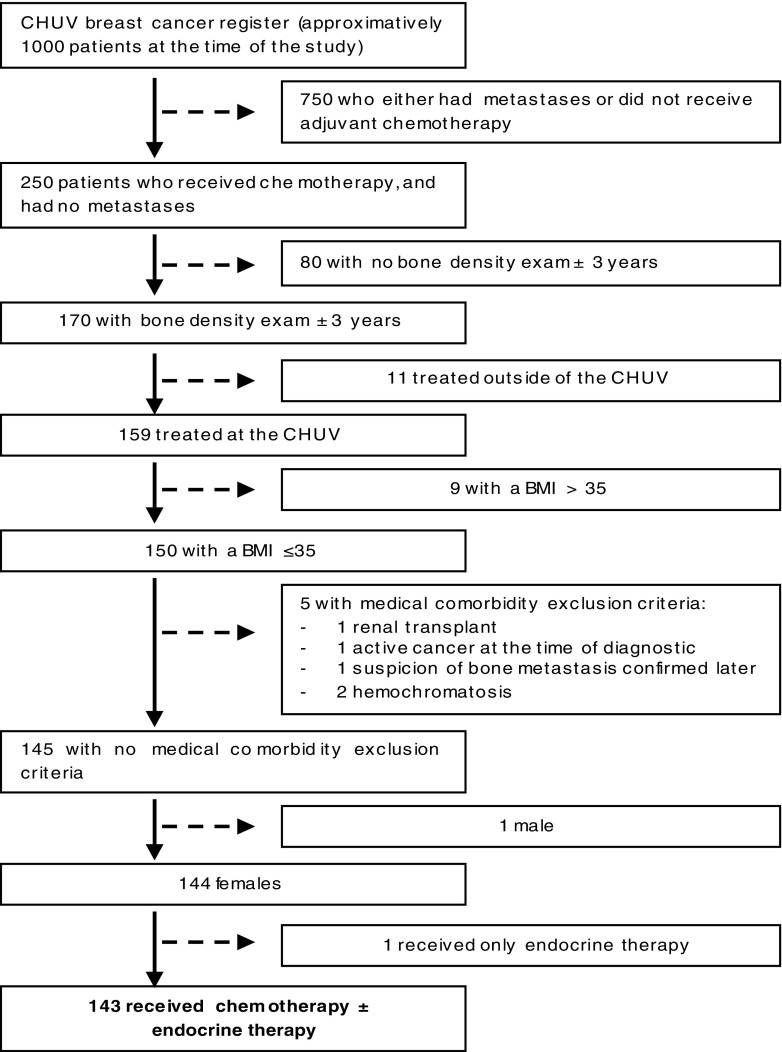
Exclusion process, CHUV, Centre Hospitalier Universitaire Vaudois: BMI, body mass index

The baseline characteristics of the 143 included women are summarized in Table 2. Active comorbidities were relatively rare in the cohort. The three more frequent active comorbidities were: arterial hypertension (23 patients, 17%), hypothyroidism (12, 8%), and diabetes (4, 3%). Treatment of hypertension, hypothyroidism, and diabetes were similarly distributed among the healthy versus osteopenic/osteoporotic groups. No patient was excluded after analysis (Fig. 1).

**Table 2:** Characteristics of the patients (*n* = 143) from the data base, osteoporotic defined by a T-score < − 2.5 or pathological fracture, osteopenia defined by a T-score between − 2.5 and − 1, normal defined by a T-score > − 1. Degraded microarchitecture defined by a TBS score < 1.2, a partially degraded microarchitecture is defined by a TBS score between 1.2 and 1.35, a normal microarchitecture is defined by a TBS score > 1.35

Characteristics	Values
Age at the start of chemotherapy (years), mean ± SD	52.13 ± 12.47
T-score	
Osteoporotic, *n* (%)	33 (32)
Osteopenia, *n* (%)	64 (45)
Normal, *n* (%)	46 (23)
TBS, mean ± SD	1.33 ± 0.12
Degraded microarchitecture, *n* (%)	20 (15)
Partially degraded microarchitecture, *n* (%)	49 (35)
Normal, *n* (%)	70 (50)
Hormonal status at the start of chemotherapy	
Premenopausal, *n* (%)	78 (55)
Postmenopausal, *n* (%)	60 (42)
Undetermined, *n* (%)	5 (3)
BMI at the start of chemotherapy (kg/m^2^), mean ± SD	24.47 ± 4.05
G-CSF administration during 1st of chemotherapy, *n* (%)	47 (33)
Infections during 1st cycle of chemotherapy, *n* (%)	43 (30)

### Blood counts before chemotherapy

**Fig. 2:**
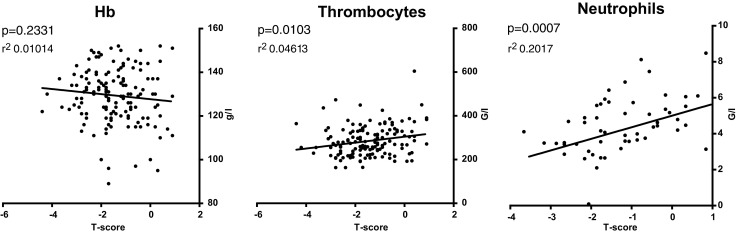
Blood cell counts according to T-score values. As BMD (bone marrow density) decreases neutrophils and thrombocytes also decrease (linear regression, *p* < 0.05), Hb ns

The blood counts before chemotherapy were analyzed according to T-score and TBS values. Our results indicate that an increase of one point in the T-score is associated with an increase of 13.51 G/l platelets and 0.644 G/l neutrophils within our cohort (Fig. 2).

No significant trends were found for the association of blood counts and TBS score before chemotherapy. Subgroup analysis using menopausal status (premenopausal and postmenopausal) did not attain a significant association between T-score and blood counts within our cohort.

### Blood counts after first cycle of chemotherapy

The evolution of the blood counts during the first cycle of chemotherapy was analyzed. Toxicity of chemotherapy was coded according to risk of febrile neutropenia as described in the methods section.

The average day of leucocyte nadir was 9.9 ± 4.2 days.

An increase of one point in TBS correlates with a decrease of 57% of the mean day count on the time for leucocyte nadir with a *p* value of 0.004 (Table 3), such that average day of leucocyte nadir was 10 days for the degraded microarchitecture group and 9.82 days for the normal microarchitecture group.

**Table 3:** A Poisson regression multivariate, TBS, B Poisson regression multivariate, T-score

A				
TBS, multivariate (Poisson regression) *n* = 139	Day of leucocyte nadir			
	Risk ratio	2.5%	97.5%	*p* value
TBS	0.43	0.24	0.76	0.0039
Age at the start of chemotherapy	0.99	0.99	1.00	0.05
G-CSF administration	0.84	0.75	0.95	0.01
Toxicity of the chemotherapy	1.04	1.02	1.06	0.000046
B				
T-score, multivariate (Poisson regression) *n* = 143	Day of leucocyte nadir			
	Risk ratio	2.5%	97.5%	*p* value
T-score	1.00	0.95	1.05	0.91
Age at the start of chemotherapy	1.00	0.99	1.00	0.45
G-CSF administration	0.84	0.74	0.95	0.0043
Toxicity of the chemotherapy	1.03	1.02	1.05	0.0002

### Rate of infection after first cycle of chemotherapy

From our analysis, rate of infection was also significantly correlated with the T-score with an odd ratio of 2.08 and a *p* value of 0.000989. TBS values did not significantly correlate with the rate of infection (Table 4).

**Table 4:** A logistical regression multivariate, T-score, B logisitical regression multivariate, TBS

A				
T-score, multivariate (logistical regression) *n* = 143	Infections			
	Odds ratio	2.5%	97.5%	*p* value
T-score	2.08	1.37	3.28	0.00099
Age at the start of chemotherapy	1.01	0.98	1.05	0.43
G-CSF administration	3.94	1.74	9.25	0.0012
Toxicity of the chemotherapy	0.83	0.71	0.96	0.02
B				
TBS, multivariate (logistical regression) *n* = 139	Infections			
	Odds ratio	2.5%	97.5%	*p* value
TBS	10.08	0.18	654.67	0.27
Age at the start of chemotherapy	1.00	0.96	1.04	0.98
G-CSF administration	4.00	1.79	9.25	0.00089
Toxicity of the chemotherapy	0.86	0.73	0.98	0.04

## Discussion

The results of our retrospective study indicate that, before chemotherapy, a higher T-score is associated with a higher neutrophil and thrombocyte count. After the first cycle of chemotherapy, our results suggest that a higher TBS score significantly correlates with a faster drop of the leucocyte count, and that a higher T-score correlates with a higher risk of infection.

There are several published studies describing a link between osteoporosis and overall lower blood counts in postmenopausal women or the elderly [30–32]. Of note, a recent study has found a paradoxical increase in the neutrophil counts of osteoporotic elderly men while monocyte and lymphocyte counts were decreased [33]. We chose to analyze our data to see if the same pattern was found. It is important to note that our patients were younger (52 years in mean), leaner (no BMI > 35), were not free of disease, as the breast cancer diagnosis was recently given, and they had recently recovered from lumpectomy/mastectomy. However, we excluded all patients with metastatic disease, making a systemic repercussion of local breast cancer less likely. We thus consider that prior to chemotherapy our cohort was subject to the minor hematopoietic stress of surgery, which also added heterogeneity to hemoglobin values given differences in blood loss during the procedure. Congruent with the literature, our analysis found a positive association between T-score and both thrombocyte and neutrophil counts. Given their very short half-life in circulation, neutrophils best reflect the ongoing function of the marrow upon stress hematopoiesis.

All previously published data refers to elderly or postmenopausal cohorts. Subgroup analysis within our cohort did not find a significant association within the postmenopausal subgroup, probably due to the reduced number of postmenopausal patients (*n* = 60). When comparing blood counts with TBS score instead of T-score, no significant association was observed. This difference may be explained by the fact that BMD evaluates the total bone mineral content, and TBS the organization of trabeculae. Thus, our data demonstrates an association between osteoporosis and lower blood counts in a younger cohort than previously published, showing for the first time decreased thrombocyte and neutrophil counts in osteoporotic women subject to a minor hematopoietic stress. Specifically, we observed an average minimum difference of 20 G/l on the thrombocyte count and 1.0 G/l on the neutrophil count between patients with normal bone and osteoporotic bone.

As bone health affects the initial blood counts and as osteoporosis is partially reversible, our study suggests that prompt treatment of osteoporosis may be beneficial to patients undergoing chemotherapy to minimize the hematopoietic toxicity. Therefore, bone assessment may be indicated before or strictly at the start of chemotherapy. Injectable antiresorptive treatments (bisphosphonates, denosumab) very rapidly reduce the activity of osteoclasts. Our work should thus prompt further studies on (i) the effect of antiresorptive treatments on complete blood counts in non-oncological patients and (ii) the pertinence of early osteoporotic treatment in oncological patients. Retrospective studies could address whether treatment of osteoporosis normalizes blood counts. The pertinence to the oncological settings should be tested by a randomized controlled study with early administration of bisphosphonates already approved for multiple myeloma or bone metastatic disease.

Regarding blood counts after the first cycle of chemotherapy, our analysis suggests that a healthier bone is associated with a more rapid drop in the total leucocyte count, or in other words, that osteoporosis slows down the leucocyte drop immediately after chemotherapy. This result is unexpected. The osteoblasts and the perivascular cells have been shown to have a supportive effect on hematopoiesis. In the osteoporotic bone, both the osteoblast count and microvasculature are reduced and the adipocyte load increased. Therefore, we had expected to see a negative effect of osteoporosis on blood counts post-chemotherapy, as it has been described in homeostatic conditions prior to chemotherapy. Our results could have different interpretations.

The osteoporotic microenvironment may indeed be actively protective to the hematopoietic progenitors immediately after chemotherapy. Alternative explanations include that the adipocytes within the bone marrow might mediate a non-specific protective effect by facilitating delayed drug release. In fact, obesity is associated with lower toxicity during chemotherapy for gynecological cancers in part due to the delayed drug release for drugs with high distribution volumes [34, 35]. Within the bone marrow, increased adipocytic mass could thus possibly translate into a lower bolus dose but a longer exposition time upon chemotherapy, especially for chemotherapy agents with a high volume of distribution. This hypothesis is compatible with the increased adipocytic content of the marrow after chemotherapy, a phenomenon recently characterized in vivo through magnetic resonance imaging upon carboplatin with paclitaxel treatment for ovarian cancer [36].

Additionally, we cannot exclude a sampling bias. Since the depth of the leucocyte drop was not significantly different between osteoporotic and non-osteoporotic patients, even though non-osteoporotic patients had significantly higher blood counts before chemotherapy, the earlier nadir of non-osteoporotic patients may just reflect a faster rate of hematological recovery in patients with healthier bone. Our results should prompt further studies in a cohort with more abundant data points for the blood count recovery.

Finally, we observed a positive association between T-score and the rate of infection. This result is also unexpected, but it is congruent with the slower drop of leucocytes in osteoporotic women, which could indicate a protective role of the osteoporotic microenvironment for infection. We could not however measure the duration of neutropenia and therefore could not test if a slower fall is associated with a shorter period of neutropenia.

Several sources of bias could also explain the observed outcome for infection. The quality of the data was lower for the qualitative analysis of infection than for the quantitative analysis of blood counts. First, the G-CSF treatment was only coded yes/no, as information was not always available to know whether it was given as primary or secondary prophylaxis of severe neutropenia. Second, the infections were recorded but no distinction was made on the severity. Of note, age was not a significant explicative variable anymore in the multivariate analysis for rate of infection. It is thus likely that other variables influence the infection rate in our cohort.

It is interesting to note that hematopoietic parameters at start correlate only with BMD and later only with TBS. Those results could be simply explained by a lack of data to reach significance with both parameters. However, a biological explanation is also possible. Spine TBS is significantly correlated with measurements of volumetric densities, cortical thickness, and whole bone stiffness at the radius and at the tibia [16]. These findings were assessed using transiliac bone biopsies, and suggest that TBS is capable of mapping the structure of trabecular bone [37]. Moreover, the correlation between LS-BMD and TBS is weak (*r* = 0.32), indicating that the two parameters were measuring different skeletal properties [37]. Conversely, LS-BMD and hip BMD were highly correlated (*r* = 0.72). The difference between BMD and TBS observed in our study could thus be explained by different niches being evolved in homeostatic hematopoiesis and stress hematopoiesis as TBS and BMD describe different bone components.

Other limitations of our cohort should be addressed. A higher number of patients might have been necessary to draw more conclusive results. However, extension of the study period would have brought more variability in the treatment options, as the treatment of breast cancer is evolving quickly. From the included patients of the “Centre du Sein,” a significant number (*n* = 80) did not have BMD and were therefore excluded. Also, a low number of postmenopausal women (*n* = 60) were part of the cohort, which limited subgroup analysis. Unfortunately, we did not have differential blood count for all patients, which most likely explains why some results including neutrophil counts post-chemotherapy did not reach statistical significance. Furthermore, due to limited blood counts, no recovery curve was calculable. Other parameters such as delay of the second cycle of chemotherapy and need for growth factors to prompt blood recovery were too rare to permit meaningful statistical analysis (one chemotherapy delayed, 19 patients received G-CSF because of low blood count).

Nevertheless, our overall observations underline that, as predicted by recent research on the bone marrow hematopoietic stem cell niche, differences in the osteoporotic bone microenvironment translate into altered dynamics upon hematopoietic stress. As other authors, we observed a positive correlation between T-score and blood counts in homeostasis or minor hematopoietic stress. After the first cycle of chemotherapy, a strong stressor for the bone marrow, we observed a slower fall of the leucocyte count in osteoporotic patients. Further studies are needed to clarify how bone physiopathology affects human hematopoiesis in different contexts both during homeostasis and stress hematopoiesis.

## Conclusion

The results of our retrospective monocentric study indicate that, before chemotherapy, a higher T-score is associated with a higher count in neutrophils and thrombocytes. This correlation was specific for T-score; no trend was observed when TBS was considered. No significant association was observed for the hemoglobin in our post-surgery cohort.

After the first cycle of chemotherapy, our results suggest that a higher TBS significantly correlates with a faster drop on the leucocyte count, and that a higher T-score correlates with a higher risk of infection. The rate of hematological recovery was not measurable due to insufficient data points. Blood counts following chemotherapy suggest that the healthier the bone, the earlier the lowest leucocyte count value.

Further studies are needed to better understand the kinetics of blood cell count recovery after chemotherapy as related to bone health.

## References

[1] Hoffbrand V, Moss P (2011) Essential haematology: includes desktop edition, 6th revised edn. Wiley-Blackwell, Malden

[2] Aguila HL, Rowe DW (2005). Skeletal development, bone remodeling, and hematopoiesis. Immunol Rev.

[3] Morrison SJ, Scadden DT (2014). The bone marrow niche for haematopoietic stem cells. Nature.

[4] Adler BJ, Kaushansky K, Rubin CT (2014). Obesity-driven disruption of haematopoiesis and the bone marrow niche. Nat Rev Endocrinol.

[5] Coşkun S, Chao H, Vasavada H, Heydari K, Gonzales N, Zhou X, de Crombrugghe B, Hirschi KK (2014). Development of the fetal bone marrow niche and regulation of HSC quiescence and homing ability by emerging osteolineage cells. Cell Rep.

[6] Calvi LM, Adams GB, Weibrecht KW, Weber JM, Olson DP, Knight MC, Martin RP, Schipani E, Divieti P, Bringhurst FR, Milner LA, Kronenberg HM, Scadden DT (2003). Osteoblastic cells regulate the haematopoietic stem cell niche. Nature.

[7] Bowers M, Zhang B, Ho Y, Agarwal P, Chen CC, Bhatia R (2015). Osteoblast ablation reduces normal long-term hematopoietic stem cell self-renewal but accelerates leukemia development. Blood.

[8] Taichman RS, Emerson SG (1998). The role of osteoblasts in the hematopoietic microenvironment. Stem Cells Dayt Ohio.

[9] Taichman RS, Emerson SG (1994). Human osteoblasts support hematopoiesis through the production of granulocyte colony-stimulating factor. J Exp Med.

[10] Visnjic D, Kalajzic Z, Rowe DW, Katavic V, Lorenzo J, Aguila HL (2004). Hematopoiesis is severely altered in mice with an induced osteoblast deficiency. Blood.

[11] Naveiras O, Nardi V, Wenzel PL, Hauschka PV, Fahey F, Daley GQ (2009). Bone-marrow adipocytes as negative regulators of the haematopoietic microenvironment. Nature.

[12] Ambrosi TH, Scialdone A, Graja A, Gohlke S, Jank AM, Bocian C, Woelk L, Fan H, Logan DW, Schürmann A, Saraiva LR, Schulz TJ (2017). Adipocyte accumulation in the bone marrow during obesity and aging impairs stem cell-based hematopoietic and bone regeneration. Cell Stem Cell.

[13] Zhu R-J, M-Q W, Li Z-J (2013). Hematopoietic recovery following chemotherapy is improved by BADGE-induced inhibition of adipogenesis. Int J Hematol.

[14] Zhou BO, Yu H, Yue R, Zhao Z, Rios JJ, Naveiras O, Morrison SJ (2017). Bone marrow adipocytes promote the regeneration of stem cells and haematopoiesis by secreting SCF. Nat Cell Biol.

[15] Mattiucci D, Maurizi G, Izzi V, Cenci L, Ciarlantini M, Mancini S, Mensà E, Pascarella R, Vivarelli M, Olivieri A, Leoni P, Poloni A (2017). Bone marrow adipocytes support hematopoietic stem cell survival. J Cell Physiol.

[16] Harvey NC, Glüer CC, Binkley N, McCloskey EV, Brandi ML, Cooper C, Kendler D, Lamy O, Laslop A, Camargos BM, Reginster JY, Rizzoli R, Kanis JA (2015). Trabecular bone score (TBS) as a new complementary approach for osteoporosis evaluation in clinical practice. Bone.

[17] Bazzocchi A, Ponti F, Diano D, Amadori M, Albisinni U, Battista G, Guglielmi G (2015). Trabecular bone score in healthy ageing. Br J Radiol.

[18] Sambrook P, Cooper C (2006). Osteoporosis. Lancet Lond Engl.

[19] Devlin MJ, Rosen CJ (2015). The bone-fat interface: basic and clinical implications of marrow adiposity. Lancet Diabetes Endocrinol.

[20] Rodríguez JP, Garat S, Gajardo H (1999). Abnormal osteogenesis in osteoporotic patients is reflected by altered mesenchymal stem cells dynamics. J Cell Biochem.

[21] Duque G (2008). Bone and fat connection in aging bone. Curr Opin Rheumatol.

[22] Shih TT-F, Chang C-J, Hsu C-Y, Wei SY, Su KC, Chung HW (2004). Correlation of bone marrow lipid water content with bone mineral density on the lumbar spine. Spine.

[23] Di Iorgi N, Mo AO, Grimm K, Wren TAL, Dorey F, Gilsanz V (2010). Bone acquisition in healthy young females is reciprocally related to marrow adiposity. J Clin Endocrinol Metab.

[24] Fraenkel M, Novack V, Liel Y, Koretz M, Siris E, Norton L, Shafat T, Shany S, Geffen DB (2013). Association between bone mineral density and incidence of breast cancer. PLoS One.

[25] Hadji P, Gnant M, Body JJ, Bundred NJ, Brufsky A, Coleman RE, Guise TA, Lipton A, Aapro MS (2012). Cancer treatment-induced bone loss in premenopausal women: a need for therapeutic intervention?. Cancer Treat Rev.

[26] Cameron DA, Douglas S, Brown JE, Anderson RA (2010). Bone mineral density loss during adjuvant chemotherapy in pre-menopausal women with early breast cancer: is it dependent on oestrogen deficiency?. Breast Cancer Res Treat.

[27] Greep NC, Giuliano AE, Hansen NM, Taketani T, Wang HJ, Singer FR (2003). The effects of adjuvant chemotherapy on bone density in postmenopausal women with early breast cancer. Am J Med.

[28] Datta M, Schwartz GG (2013). Calcium and vitamin D supplementation and loss of bone mineral density in women undergoing breast cancer therapy. Crit Rev Oncol Hematol.

[29] Zielinski CC, Awada A, Cameron DA, Cufer T, Martin M, Aapro M (2008). The impact of new European Organisation for Research and Treatment of Cancer guidelines on the use of granulocyte colony-stimulating factor on the management of breast cancer patients. Eur J Cancer Oxf Engl 1990.

[30] Kim H-L, Cho HY, Park IY, Choi JM, Kim M, Jang HJ, Hwang SM (2011). The positive association between peripheral blood cell counts and bone mineral density in postmenopausal women. Yonsei Med J.

[31] Di Monaco M, Vallero F, Di Monaco R (2004). Total lymphocyte count and femoral bone mineral density in postmenopausal women. J Bone Miner Metab.

[32] Laudisio A, Marzetti E, Pagano F, Bernabei R, Zuccalà G (2009). Haemoglobin levels are associated with bone mineral density in the elderly: a population-based study. Clin Rheumatol.

[33] Valderrábano RJ, Lui L-Y, Lee J, Cummings SR, Orwoll ES, Hoffman AR, Wu JY, for the Osteoporotic Fractures in Men (MrOS) Study Research Group (2017). Bone density loss is associated with blood cell counts. J Bone Miner Res Off J Am Soc Bone Miner Res.

[34] Carroll J, Protani M, Walpole E, Martin JH (2012). Effect of obesity on toxicity in women treated with adjuvant chemotherapy for early-stage breast cancer: a systematic review. Breast Cancer Res Treat.

[35] Hansen J, Stephan J-M, Freesmeier M, Bender D, Button A, Goodheart MJ (2015). The effect of weight-based chemotherapy dosing in a cohort of gynecologic oncology patients. Gynecol Oncol.

[36] Cawthorn WP, Scheller EL, Learman BS, Parlee SD, Simon BR, Mori H, Ning X, Bree AJ, Schell B, Broome DT, Soliman SS, DelProposto JL, Lumeng CN, Mitra A, Pandit SV, Gallagher KA, Miller JD, Krishnan V, Hui SK, Bredella MA, Fazeli PK, Klibanski A, Horowitz MC, Rosen CJ, MacDougald OA (2014). Bone marrow adipose tissue is an endocrine organ that contributes to increased circulating adiponectin during caloric restriction. Cell Metab.

[37] Shevroja E, Lamy O, Kohlmeier L, Koromani F, Rivadeneira F, Hans D (2017). Use of trabecular bone score (TBS) as a complementary approach to dual-energy X-ray absorptiometry (DXA) for fracture risk assessment in clinical practice. J Clin Densitom Off J Int Soc Clin Densitom.

